# The Relations Between HSG Proven Tubal Occlusion, Stimulated Intrauterine Insemination and Pregnancy Rate

**DOI:** 10.4274/balkanmedj.2016.0289

**Published:** 2017-01-05

**Authors:** Gonca Yetkin Yıldırım, Ahu Orta Korkut, Nadiye Köroğlu, Lale Susan Türkgeldi

**Affiliations:** 1 Department of Obstetrics and Gynaecology, Kanuni Sultan Süleyman Training and Research Hospital, İstanbul, Turkey; 2 Department of Obstetrics and Gynaecology, Mardin Women and Children Hospital, Mardin, Turkey

**Keywords:** Unilateral tubal occlusion, Pregnancy, ovulation induction, intrauterine insemination

## Abstract

**Background:**

Tubal factor infertility is one of the main causes of female infertility. Although its sensitivity is low, hysterosalpingography (HSG) is remains the first-line method for evaluating tubal patency.

**Aims:**

To compare pregnancy rates in patients with HSG proven proximal or distal unilateral tubal occlusion, and unexplained infertility undergoing both controlled ovarian stimulation (COS) and intrauterine insemination (IUI).

**Study Design:**

Case control study.

**Methods:**

In total, 237 patients undergoing ovulation induction (OI) with gonadotropins and IUI were divided into two groups and evaluated. Study group consisted 59 patients with HSG proven unilateral tubal pathology, and 178 patients with unexplained infertility taken as control subjects. Cumulative pregnancy rate was the primary endpoint.

**Results:**

Cumulative pregnancy rates after three cycles of OI and IUI were 15.25% in study group and 20.79% in control group. Pregnancy rates between two groups were not statistically significant. Although, pregnancy rates in patients with proximal tubal occlusion (21.8%) were higher than in those with distal tubal occlusion (7.4%), the difference was not statistically significant.

**Conclusion:**

Our study data shows that, regardless of the HCG proven occlusion area, COS and IUI might be a preferred treatment modality in patient with unilateral tubal occlusion.

Evaluation of tubal disease is an integral part of the infertility workup in many centres offering fertility treatment around the world. Although laparoscopic evaluation of fallopian tubes using methylene blue is considered the gold standard, tubal patency is most often evaluated with a hysterosalpingogram (HSG) (1). HSG is a minimally invasive and low-cost outpatient procedure with a reported sensitivity and specificity for detecting tubal pathology of 65% and 83%, respectively (2). The relatively low sensitivity of HSG with respect to its specificity is due to its inability to differentiate between transient and pathological tubal obstructions. Another drawback of HSG is that even if tubal patency is demonstrated, information about the function of the tube cannot be obtained. In patients with hysterosalpingographic findings of a bilateral tubal obstruction, the patient is either offered a laparoscopic evaluation for tubal patency and pelvic pathology and subsequent reconstructive surgery or is referred directly for in vitro fertilisation (IVF) treatment. For those with unilateral tubal patency laparoscopic surgery, direct referral of the patient for IVF or ovulation induction (OI) and intrauterine insemination (IUI) has been suggested as an acceptable approach (3). However, OI and IUI seems to be the preferred initial treatment approach for such patients in many centres due to its non-invasive nature and lower cost compared to IVF or tubal surgery. Several studies have reported similar pregnancy rates among patients with unexplained infertility and unilateral tubal patency following OI and IUI (3,4). Although patients with proximal tubal occlusion appear to have more favourable pregnancy outcomes than those with a distal tubal obstruction, a statistically significant difference has not been demonstrated (5).

In this study we aimed to present our data on pregnancy rates after OI cycles with gonadotropins and IUI in patients with unilateral tubal occlusion and unexplained infertility, and to determine whether or not there is a significant prognostic difference in pregnancy rates between cases with proximal and distal tubal obstruction evident on an HSG.

## MATERIALS AND METHOD

The medical records of 237 patients who had undergone ovarian stimulation with gonadotropins and IUI with a diagnosis of unexplained infertility or unilateral tubal occlusion in the infertility department between January 2003 and December 2012 were reviewed retrospectively. The approval of the institutional ethics committee was obtained. The infertility workup included a detailed history (including age, duration of infertility, medical history, coital frequency, prior pelvic surgeries), physical examination, gynaecological examination, cycle day 3 follicle-stimulating hormone (FSH), estradiol, thyroid stimulating hormone, luteinizing hormone, prolactin levels and day 21 progesterone levels, histerosalpingography reports and a spermiogram.

Patients between 21 and 38 years of age, with normal day 3 hormone and day 21 progesterone levels, transvaginal ultrasonographic imaging and spermiogram parameters, and findings of a normal uterine cavity and no hydrosalpinges on the HSG were included in the study. All patients were examined by one of two infertility specialists (G. Y. or İ. P.) and were treated with the same induction and IUI protocol.

The patients were categorized into two groups. The study group consisted of patients with hysterosalpingographic evidence of only one-sided tubal patency and the control group included patients with evidence of bilateral tubal patency. The unilateral tubal patency group was further subdivided into those with proximal (n=32) or distal tubal occlusion (n=27).

The patients were treated with recombinant FSH (GonalF; Serono. Zug, Switzerland) or urinary gonadotropins (Menogon; Fering, Copenhagen, Denmark) starting on day 3 of the menstrual cycle. The starting gonadotropin dose in the first cycle was 37.5 IU and subsequent dosages were adjusted according to the individual follicular response monitored every 2 to 3 days. Ovulation was triggered with urinary human chorionic gonadotropin (hCG) (Chorigon: Teva, Petah Tigva, Israel or Pregnyl; Organon, Oss, the Netherlands) or 250 micrograms of recombinant hCG (Ovitrelle; Serono). Sperm samples were prepared using the swim-up technique and insemination was performed with a soft catheter 36 hours after the hCG injection. A serum beta hCG was obtained 15 days following the hCG trigger, and those with a positive pregnancy test were followed up until foetal heartbeats were visible on ultrasound. No patients were lost to follow-up. All couples were offered three cycles of OI and IUI unless pregnancy had occurred in the previous cycles. The primary outcome measure was the cumulative pregnancy rate.

Using Berker’s study as a guide, a power analysis was performed and a sample size of 255 was estimated to be required to obtain a power of 80% for an approximately 18.4% difference in cumulative pregnancy rates. Since our sample size is lower than this estimated number, a type II error could not be excluded for this parameter. It is difficult to recruit such a number of patients in a single centre, therefore we aimed to analyse a 9-year cohort of all patients in our centre, which could be included in future meta-analyses.

### Statistical analyses

Statistical analyses were performed using the NCSS (Number Cruncher Statistical System) Statistical Software 2007 (Utah, USA) package program. The Mann-Whitney U test was used for the comparison of non-parametric continuous data and the chi-square and Fisher’s exact tests were used for categorical data. P<0.05 was considered to be statistically significant.

## RESULTS

The study group consisted of 59 patients diagnosed with unilateral tubal occlusion who underwent a total of 165 cycles of OI and IUI. The control group consisted of 178 patients with unexplained infertility who underwent 490 cycles of OI and IUI.

A comparison of clinical parameters is presented in Table 1. There were no statistically significant differences between the mean age, duration of infertility and parity between the study and control groups. However, secondary infertility was significantly higher in the study group than in the control group (p=0.021). The remaining clinical parameters were similar between the two groups.

A significantly higher rate of previous abdominal surgery or ectopic pregnancy was detected in the study group than in the control group (p=0.0001 and p=0.004, respectively). A history of PID was found in only one patient in the study group and in no patients in the control group.

The pregnancy rate after OI and IUI in patients with unilateral tubal occlusion was 15.25%, and it was 20.79% in the unexplained infertility group. The difference between the two groups with respect to pregnancy rate was not statistically significant (p=0.352). The pregnancy rates of patients with proximal tubal occlusion and distal tubal occlusion were 21.87% and 7.4%, respectively. The difference between the two groups was not statistically significant (p=0.183) (Table 2).

A multiple logistic regression analysis was done to evaluate factors that affect pregnancy outcome as presented in Table 3.

## DISCUSSION

The results of the present study indicate that patients with findings of unilateral tubal blockage on a HSG have similar pregnancy rates (15.25%) to patients with unexplained infertility (20.79%) following OI with gonadotropins and IUI. Although pregnancy rates in cases with proximal tubal blockage (21.8%) were higher than in those with distal obstruction (7.4%), the difference did not reach statistical significance.

Mol et al. (6) failed to find a difference in fertility outcomes between patients with hysterosalpingographic findings of unilateral tubal pathology and bilateral tubal patency. Therefore they suggested the same treatment approach to these two groups of patients. However, the presence of unilateral tubal disease may still be a risk factor for contralateral tubal disease that is not evident as a pathological finding on an HSG, which may cause lower pregnancy rates than expected. The efficacy of HSG in diagnosing functional or anatomical tubal pathologies as peritubal adhesions is reported to be low (7). Moreover, the false negative rate of HSG for the diagnosis of bilateral tubal patency is reported to be 10% (8). In a subgroup of patients with unexplained infertility there may be an underlying tubal functional pathology, including a lack of coordinated muscular contractions or ciliary activity, which cannot be demonstrated by HSG or laparoscopy. Therefore, patients with unexplained infertility may not be an ideal control group of patients to compare with those with unilateral tubal pathology in order to demonstrate the effect of tubal pathology on fertility rates. That being said, the comparison of pregnancy rates between the two groups of infertile patients is helpful in deciding whether treatment with OI and IUI is worth considering in patients with unilateral tubal patency.

In line with the findings of the present study, Farhi et al. (3) demonstrated similar cumulative pregnancy rates in patients with unilateral tubal patency (30.9%) and those with unexplained infertility (42.6%) after three cycles of controlled ovarian hyperstimulation with gonadotropins and IUI. They also found unilateral mid-distal or distal obstruction to be associated with lower pregnancy rates than those with proximal obstruction, although this difference did not reach statistical significance. They suggested controlled ovarian hyperstimulation and IUI as the initial treatment plan for those with proximal obstruction. For patients with mid-distal or distal obstruction, either surgical correction of tubal pathology or direct referral of patients for IVF treatment was recommended. Similarly, Ebrahimi et al. (4) stated that unilateral tubal blockage had no effect on the success rates of IUI in stimulated cycles with clomiphene citrate or gonadotropins.

Berker et al. (5), on the other hand, found significantly lower cumulative pregnancy rates in patients with unilateral tubal occlusion (26.3%) than in those with unexplained infertility (44.7%). However, when evaluated according to the location of the obstruction, patients with proximal blockage were found to have similar pregnancy rates to those with unexplained infertility, whereas those with distal obstruction had lower pregnancy rates. They also recommended IVF treatment instead of IUI in patients with distal obstruction.

The reported similar pregnancy rates in patients with proximal tubal occlusion on an HSG and those with unexplained infertility can in part be attributed to the fact that up to 60% of patients with unilateral tubal patency have been shown to demonstrate bilateral tubal patency after a second HSG (9). This temporary proximal tubal occlusion may be caused by a transient cornual spasm or flushing of mucus plugs or cellular debris from the proximal tube after the first HSG. The presence of a distal tubal occlusion, however, is more likely to reflect a true tubal pathology, usually occurring as the consequence of a previous salpingitis, ectopic pregnancy, abdominal surgery or appendicitis. The lack of a statistically significant difference between those with distal tubal obstruction and unexplained infertility indicates that a patent unilateral tube is sufficient for the occurence of a pregnancy. This is supported by the reported spontaneous pregnancy rate of 64% in patients following unilateral salpenjectomy for ectopic pregnancy (10). In patients with unilateral tubal obstruction, the efficacy of surgical intervention or IVF with respect to OI and IUI is not clear, since no studies comparing these treatment modalities have been conducted.

There are some limitations to this study. First, it was a retrospective study. Second, the sample size was small and the number of patients for each study arm necessary for confirmation of statistical significance could not be reached. Large-scale prospective randomized studies should be planned to establish an appropriate management protocol for unilateral tubal factor infertility.

It is our opinion that it is acceptable to offer at least three trials of OI and IUI to patients with unilateral tubal obstruction demonstrated on an HSG, irrespective of the location of the occlusion, since the overall clinical pregnancy rates have been shown to be similar to those in patients with unexplained infertility and a statistically significant difference has not yet been demonstrated between those with distal or proximal tubal obstruction and unexplained infertility.

## Figures and Tables

**Table 1:**
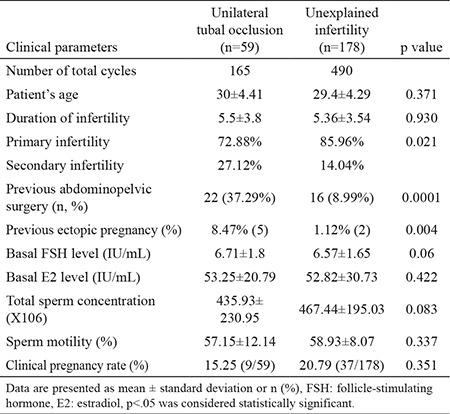
Clinical parameters of unilateral tubal occlusion and unexplained infertility

**Table 2:**
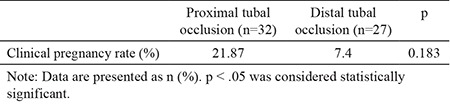
Comparison of clinical pregnancy rates in patients with proximal and distal tubal occlusion

**Table 3:**
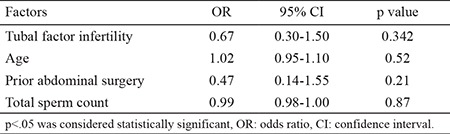
Predictor factors of pregnancy according to the results of multivariate analysis
